# Inflammatory Biomarkers Are Inaccurate Indicators of Bacterial Infection on Admission in Patients With Acute Exacerbation of Chronic Obstructive Pulmonary Disease—A Systematic Review and Diagnostic Accuracy Network Meta-Analysis

**DOI:** 10.3389/fmed.2021.639794

**Published:** 2021-11-18

**Authors:** Piroska Pázmány, Alexandra Soós, Péter Hegyi, Dóra Dohos, Szabolcs Kiss, Zsolt Szakács, Andrea Párniczky, András Garami, Zoltán Péterfi, Zsolt Molnár

**Affiliations:** ^1^Medical School, Institute for Translational Medicine, University of Pécs, Pécs, Hungary; ^2^Department of General Medicine and Pulmonology, Heim Pál National Institute for Pediatrics, Budapest, Hungary; ^3^Doctoral School of Clinical Medicine, University of Szeged, Szeged, Hungary; ^4^János Szentágothai Research Center, University of Pécs, Pécs, Hungary; ^5^Centre for Translational Medicine, Semmelweis University, Faculty of Medicine, Budapest, Hungary; ^6^Department of Gastroenterology, Heim Pál National Institute for Pediatrics, Budapest, Hungary; ^7^Department of Internal Medicine, Medical School, University of Pécs, Pécs, Hungary; ^8^Department of Anesthesiology and Intensive Therapy, Medical Faculty, Poznan University for Medical Sciences, Poznan, Poland; ^9^Department of Anaesthesiology and Intensive Therapy, Semmelweis University, Faculty of Medicine, Budapest, Hungary

**Keywords:** chronic objective pulmonary disease, bacterial infection, acute exacerbation, inflammatory biomarker, diagnostic accuracy, meta-analysis, microbiological culture

## Abstract

**Introduction:** The value of inflammatory biomarkers in the diagnosis of bacterial infection induced acute exacerbation of chronic obstructive pulmonary disease (AECOPD) is currently unclear. Our objective was to investigate the diagnostic accuracy of on-admission inflammatory biomarkers in differentiating bacterial origin in AECOPD.

**Methods:** Systematic literature search was performed to include cross-sectional studies on AECOPD patients with microbiological culture results as gold standard, and at least one on-admission inflammatory biomarker determined from serum: C-reactive protein (CRP), procalcitonin (PCT), neutrophil/lymphocyte ratio, eosinophil percentage, CD64index; or sputum: neutrophil elastase, tumor necrosis factor alfa, interleukin-1-beta (IL-1b), interleukin-8, sputum color, as index tests. We ranked index tests by superiority indices in a network meta-analysis and also calculated pooled sensitivity and specificity.

**Results:** Altogether, 21 eligible articles reported data on 2,608 AECOPD patients (44% bacterial). Out of the 14 index tests, sputum IL-1b showed the highest diagnostic performance with a pooled sensitivity of 74% (CI: 26–97%) and specificity of 65% (CI: 19–93%). Pooled sensitivity for CRP and PCT were: 67% (CI: 54–77%) and 54% (CI: 39–69%); specificity 62% (CI: 52–71%) and 71% (CI: 59–79%), respectively.

**Conclusion:** Admission inflammatory biomarkers are inaccurate indicators of bacterial infection in AECOPD.

**Systematic Review Registration:** https://www.crd.york.ac.uk/prospero/#myprospero, identifier: 42020161301.

## Background

Chronic obstructive pulmonary disease (COPD) is characterized by chronic inflammation of the airways, parenchyma and pulmonary vasculature (1–4). COPD has been reported as being the third leading cause of deaths worldwide in 2016, with around 3 million deaths annually (4).

Acute exacerbation of COPD (AECOPD) is a serious event often requiring hospitalization, including admission to intensive care unit. Frequent exacerbations may lead to disease progression. The Anthonisen criteria for AECOPD comprises: the acute worsening in the patient's baseline dyspnea, cough and/or sputum production beyond day-to-day variability leading to a change in medication (5). The majority—around 80% of exacerbations—are managed on an outpatient basis (3). The hospitalization rate ranges between 0.15 and 0.25 number/patients/year, with an average hospital length of stay of 8.7 days and an overall mortality of 8% (6, 7). Published data suggest that 50–70% of exacerbations are due to respiratory infections (bacterial, viral, atypical pathogens), 10% to environmental pollution (depending on season and geographical placement) and up to 30% are of unknown etiology (8, 9).

The role of biomarkers in COPD and AECOPD have been studied for several decades. Inflammatory biomarkers can be used as a diagnostic tool for early detection of events, or to classify subgroups. There is also wide range of studies on the prognostic ability of biomarkers to predict clinical outcomes. Most importantly the utilization of biomarkers to identify treatment indications is a highly researched area (10).

Confirming a bacterial precipitant of AECOPD is difficult on admission, but also important, as either delayed or inadequate antibiotic treatment can result in serious complications (11–14). In COPD chronic bacterial colonization is a frequently observed phenomenon. This adds to the uncertainty of diagnosing exacerbations caused by new strains of bacterial infection. A recent study by Sethi et al. found that acquisition of a new, previously unencountered strain of bacteria is associated with higher risk of progressing to exacerbation. Also new strain exacerbations were associated with higher inflammatory response compared to excerbations without documented new pathogens (10, 15).

Due to the lack of gold standard for diagnosing bacterial etiology of exacerbations, the 2020 GOLD report suggests the initiation of antibiotic therapy when the following three clinical signs are present: (1) increase in dyspnea, (2) increase in sputum volume and (3) increase in sputum purulence, or if the patient requires either invasive or non-invasive mechanical ventilation (3). Positive microbiological culture in addition to these clinical signs remains the most reliable tool to diagnose bacterial exacerbations. Microbiological testing and the identification of bacterial growth unfortunately takes considerable time (1–3 days). Therefore, in most cases, empirical antibiotic treatment is initiated and as a consequence unnecessary or inadequate antibiotic administration is common. Interestingly, a recent randomized controlled trial reported that a biomarker-based approach could reduce antibiotic exposure by 50%, without compromising outcome in patients admitted to the Emergency Department with acute respiratory complaints compared to controls in whom only conventional clinical and laboratory signs were used (16). Therefore, it is not surprising that there is an emerging interest for utilizing biomarkers to detect bacterial infection in AECOPD to aid the decision on initiating antibiotic therapy (3).

Current evidence is controversial on the reliability of inflammatory biomarkers in diagnosing bacterial infections and guiding antibiotic therapy in AECOPD (3). Our clinical question is whether it is possible to differentiate bacterial induced exacerbation of COPD on admission, using inflammatory biomarkers. Since the relative performance of inflammatory biomarkers has not been determined in its full complexity, our aim was to test and compare the diagnostic ability of serum and sputum biomarkers that have been investigated in available literature, in order to distinguish bacterial and non-bacterial AECOPD by performing a diagnostic test accuracy network meta-analysis (DTA-NMA). The DTA-NMA enables comparison of the diagnostic tests in the absence of head-to-head comparisons. Findings from our DTA-NMA might aid clinicians and guideline developers to improve diagnostics of bacterial AECOPD.

## Results

### Results of Systematic Search and Selection

**Figure 1:**
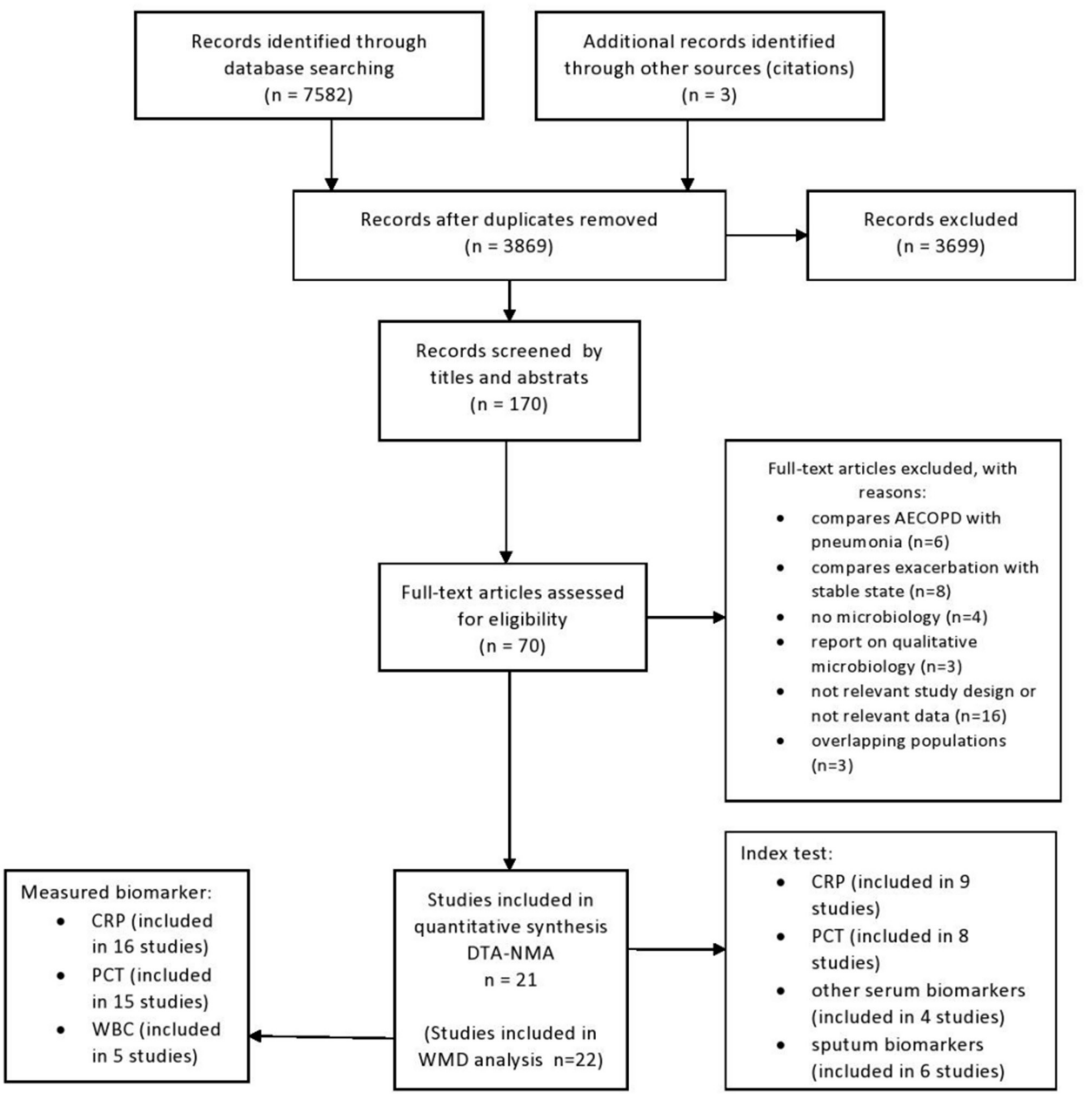
PRISMA flowchart of systematic search and selection.

Altogether 21 studies (19 full texts and two abstracts) were eligible for inclusion. PRISMA flowchart for systematic selection is presented on Figure 1. The studies reported data on a total of 2,608 AECOPD cases of which 1,142 (44%) had a positive bacterial culture. Altogether the studies evaluated 14 index tests from either serum or sputum samples. CRP and PCT were the most commonly investigated biomarkers. See details of systematic search and selection in Supplementary Table 1.

### Characteristics of the Included Studies

The characteristics of the studies included in the DTA-NMA are reported in Table 1. The results of diagnostic accuracy testing as reported in each included study is reported in Supplementary Table 2.

**Table 1:** Characteristics of included studies.

**Author, Year of publication**	**Country**	Study centers and design^**^	**Population (recruitment period and inclusion criteria)**		**Timing of sample collection**	**Reference standard (microbiological culture obtained from)**	**Index tests (cut-off value)**	**Sample size (bacterial %)**
Tanriverdi et al. (17)	Turkey	Single centerProspective cohort	Jan 01–Mar 31 2014	Hospitalized AECOPD patients	On-admission	Tracheal aspirate or sputum	CRP (91.5 mg/l) PCT (0.4 ng/ml) N/L ratio (11.5)	77 (36)
Dev et al. (18)	United Kingdom	Prospective cohort	Not reported	Hospitalized AECOPD patients	On-admission	Sputum	CRP (10 mg/l)	50 (58)
Peng et al. (19)	China	Single centerProspective cohort	Not reported	Hospitalized AECOPD patients	On-admission	Sputum	CRP (19.65 mg/l)	81 (68)
Clark et al. (20)	United Kingdom	Single centerRetrospective analysis	Between Sept 2005 and May 2008	Hospitalized AECOPD^*^ patients	On-admission	Blood, sputum, urine and nasopharyngeal swab	CRP (10 mg/l)	264 (25)
Xiong et al. (21)	China	Single centerProspective cohort	Jan 2014–Jan 2016	Hospitalized AECOPD patients	On-admission	Sputum	CRP (31.68 mg/l) PCT (0.76 ng/ml) SAA (31.28 mg/l)	78 (49)
Sethi et al. (15)	USA	Single centerProspective cohort	Jan 01 1999–Dec 31 2000	COPD outpatients, followed monthly and at exacerbations	At each clinic visit, at exacerbation	Sputum	CRP (2.37 mg/l) Sputum NE (0.76 nM) Sputum IL-8 (1.39 ng/ml) Sputum TNFa (320 pg/ml)	150 exacerbations from 46 patients (26)
Bathoorn et al. (22)	Netherlands	Single center prospective cohort	Not reported	COPD outpatients experiencing an exacerbation	At each clinic visit, at exacerbation	Sputum	CRP (2 mg/l) Sputum TNFa (30 pg/ml) Sputum MPO (12 ug/ml)	37(22)
Numbere et al. (23)	United Kingdom	Single center retrospective analysis (congress abstract)	Not reported	Hospitalized AECOPD^*^ patients	Within 24 h of admission	Sputum	CRP (50 mg/l)	122 (55)
Bafadhel et al. (24)	United Kingdom	Single centerProspective cohort	Not reported	COPD outpatients with >1 exacerbations in the preceding 12 months	At each visit	Sputum	CRP (10 mg/l) Sputum IL-1b (125 pg/ml)	148 exacerbations (75 patients) (53)
Nseir et al. (25)	France	Single centerProspective cohort	Dec 2004–June 2006	Hospitalized AECOPD patients, requiring mechanical ventilation	ICU admission, before AB therapy	Endotracheal aspirate	PCT (0.5 ng/ml)	98 (41)
Ergan et al. (26)	Turkey	Single centerProspective cohort	May 01 2007- July 31 2009	Hospitalized AECOPD patients, requiring ICU admission	ICU admission	Sputum or endotracheal aspirate or bronchoalveolar	PCT (0.25 ng/ml)	52 (31)
Falsey et al. (9)	USA	Single centerprospective cohort	Between Nov 01 and May 30 2008–2009 and 2009–2010	Hospitalized AECOPD patients	At enrollment	Blood culture, sputum Gram stain and culture, nose and throat swabs	PCT (0.25 ng/ml)	184 (17)
Chang et al. (27)	Taiwan	Single centerProspective cohort	Aug 2009–Aug 2010	COPD patients who visited ED with acute exacerbation	At Emergency Department visit	Sputum	PCT (0.5 ng/ml)	72 (42)
Chang et al. (28)	China	Single centerProspective cohort	Not reported	AECOPD^*^ outpatients	At presentation of exacerbation	Sputum	PCT (0,155 ng/ml)	45 (33)
Daubin et al. (29)	France	Single centerProspective cohort	Sept 2005–Sept 2006	Hospitalized AECOPD patients	At ICU admission	Sputum or tracheal aspirate	PCT (0,1 ng/ml)	35 (14)
Choi et al. (30)	Korea	Single centerRetrospective analysis	Jan 2011–May 2017	Hospitalized AECOPD patients	On-admission	Blood, sputum, and urine	Eosinophil (2%)	736 (42)
Qian et al. (31)	China	Single centerRandomized controlled trial	Jan–Dec 2014	Hospitalized AECOPD patients	On-admission	Sputum	CD64 index (2,5)	150 (55)
Soler et al. (32)	Spain	Single centerRandomized controlled trial	Jan 2007–May 200	Hospitalized AECOPD patients	At enrollment	Sputum	Sputum color (purulent)	41 (34)
Burley et al. (33)	United Kingdom	Multicenter prospective cohort	Nov 1999–Feb 2003	Adult patients with AECOPD	At enrollment	Sputum	Sputum color (purulent)	97 (60)
Stockley et al. (34)	United Kingdom	Single centerProspective cohort	Not reported	AECOPD outpatients	On-admission	Sputum	Sputum color (purulent)	121 (71)
Dal Negro et al. (35)	Italy	Single centerProspective cohort	Not reported	Hospitalized AECOPD patients	On-admission	Sputum	Sputum TNFa+IL-8+IL-1b (492 pg/ml, 4,811 ng/ml, 2,818 pg/ml)	124 (39)

* These studies did not identify COPD according to the GOLD (3) criteria, and AECOPD according to Anthonisen criteria (5) (Populations in studies not marked with * were defined by GOLD criteria and Anthonisen criteria). One article (15) defined AECOPD according to the guidelines of the American Thoracic Society (36). The differences in these definitions can be considered negligible in clinical practice. The included abstracts (22, 27) did not provide information on by which definition AECOPD was defined.

** *Study designs are shown according to full text articles, however in this network meta-analysis we interpreted only admission findings, therefore we considered the studies as cross sectional studies*.

*AECOPD, acute exacerbation of chronic obstructive pulmonary disease; CRP, C-reactive protein; ED, Emergency Department; ICU, intensive care unit; IL-1b, interleukin one beta; IL-8, interleukin eight; MPO, myeloperoxidase; NE, neutrophil elastase; N/L, neutrophil-lymphocyte ratio; PCT, procalcitonin; SAA, serum amyloid A; TNFa, tumor necrosis factor alfa*.

Characteristics of studies included in the additional analysis (WMD analysis) are shown in the Supplementary Table 3.

### Weighted Mean Difference Analysis of Biomarkers—Additional Analysis

To investigate the hypothesis of whether inflammatory biomarker levels differ in the bacterial and non-bacterial etiologies of AECOPD at initial presentation, we analyzed the direct comparisons of CRP, PCT, and WBC count based on the reported measurements from eligible studies. The heterogeneity of the studies is large which limits the interpretation of the results.

**Figure 2:**
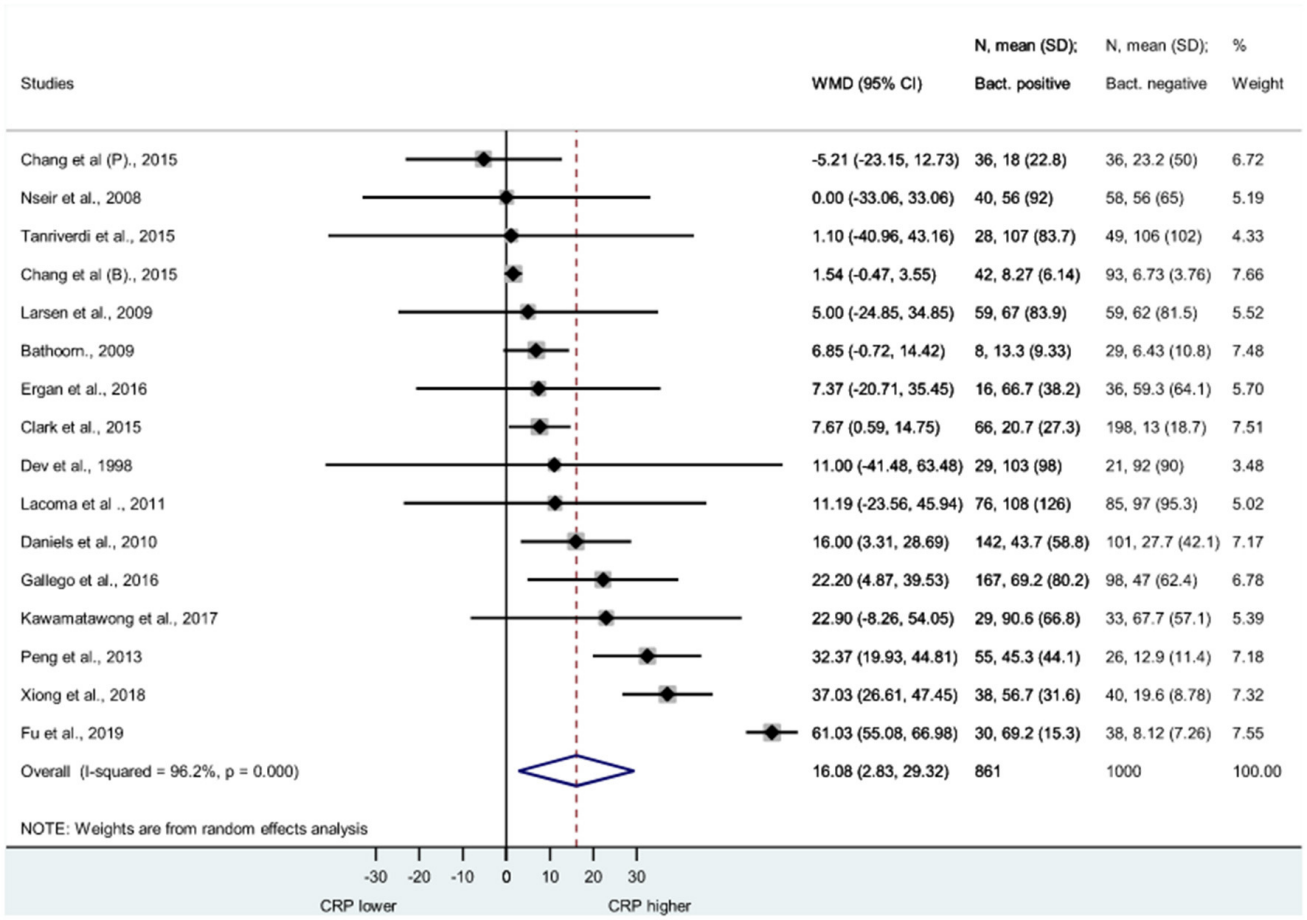
Forest plot of serum C-reactive protein. CRP was measured in AECOPD cases with bacteria positive and bacteria negative microbiological cultures. CRP was measured in (mg/l) in each study. CRP is significantly elevated in bacterial cases than non-bacterial cases. WMD = 16.06, 95% CI: 2.83–29.32, *p* = 0.017. AECOPD, acute exacerbation of chronic obstructive pulmonary disease; CRP, C-reactive protein; SD, standard deviation; WMD, weighted mean difference; CI, confidence interval.

Figure 2 shows the Forest plot of studies, illustrating the WMD of CRP (measured in mg/l in each study) between bacterial and non-bacterial AECOPD. Weighted mean of CRP proved to be significantly elevated in bacterial AECOPD compared to non-bacterial; WMD = 16.08, 95% CI: 2.83–29.32 (*p* = 0.017).

**Figure 3:**
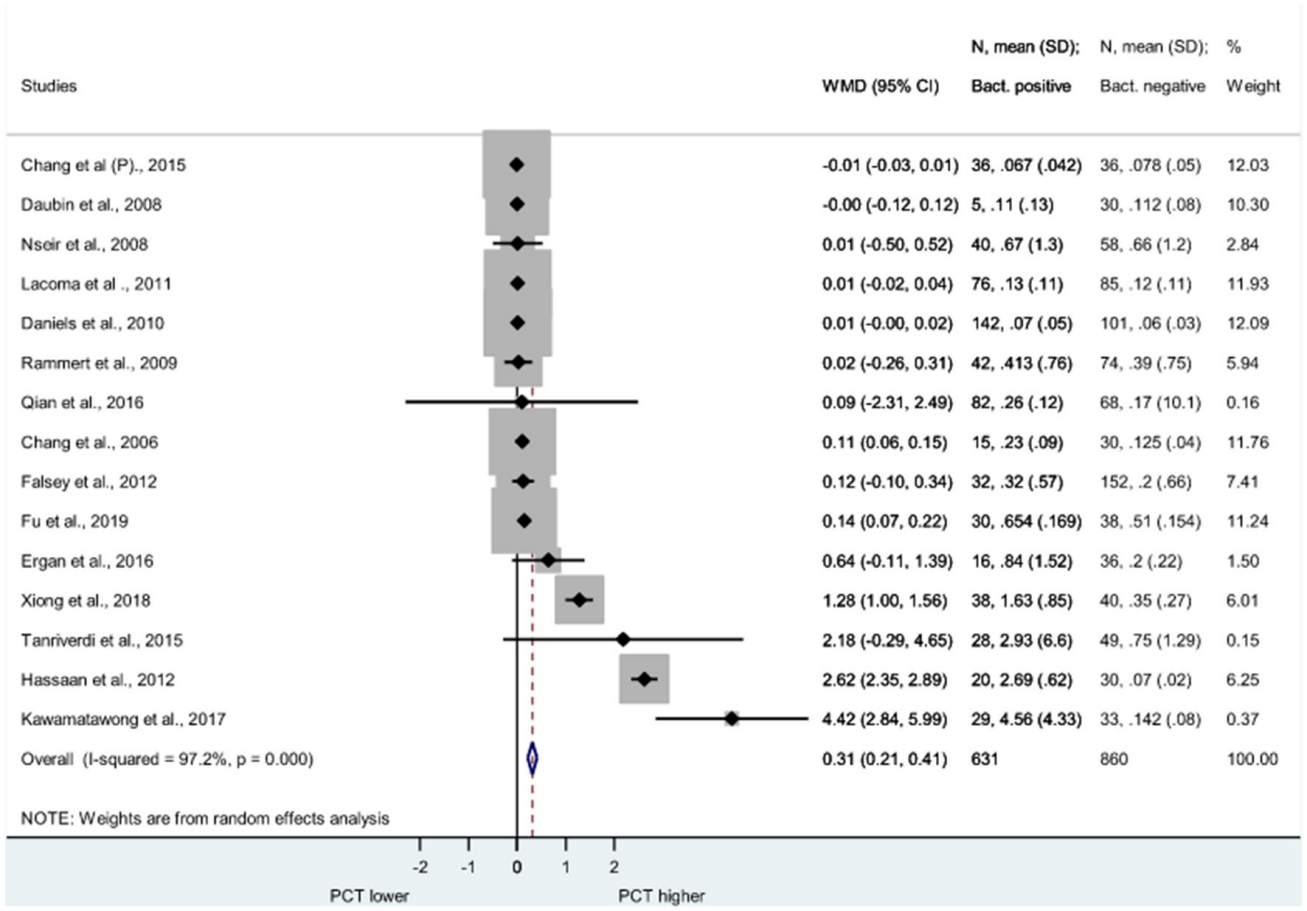
Forest plot of serum procalcitonin. PCT was measured in AECOPD cases with bacteria positive and bacteria negative microbiological cultures. PCT was measured in (ng/ml) in each study. PCT is significantly elevated in bacterial cases than non-bacterial cases. (WMD = 0.31, 95% CI: 0.21–0.41, *p* < 0.001). AECOPD, acute exacerbation of chronic obstructive pulmonary disease; PCT, procalcitonin; SD, standard deviation; WMD, weighted mean difference; CI, confidence interval.

Figure 3 demonstrates the Forest plot of PCT (measured in ng/ml in each study) in bacterial and non-bacterial AECOPD cases. PCT was also significantly higher in bacterial exacerbations than in non-bacterial cases; WMD = 0.31, 95% CI: 0.2 to −0.41 (*P* < 0.001).

Direct comparison of WBC counts (measured in 1,000/ml in each study) also showed significant elevation in bacterial AECOPD cases compared to non-bacterial cases; WMD = 1.07, 95% CI: 0.40–1.74 (*p* = 0.002). Forest plot of WMD in bacterial and non-bacterial AECOPD is presented in the Supplementary Figure 1.

As the measured biomarker levels were significantly different between bacterial and non-bacterial cases, determining a threshold and investigating the diagnostic performance of biomarkers to differentiate between the two groups seemed reasonable.

### Components of the Network in DTA-NMA

**Figure 4:**
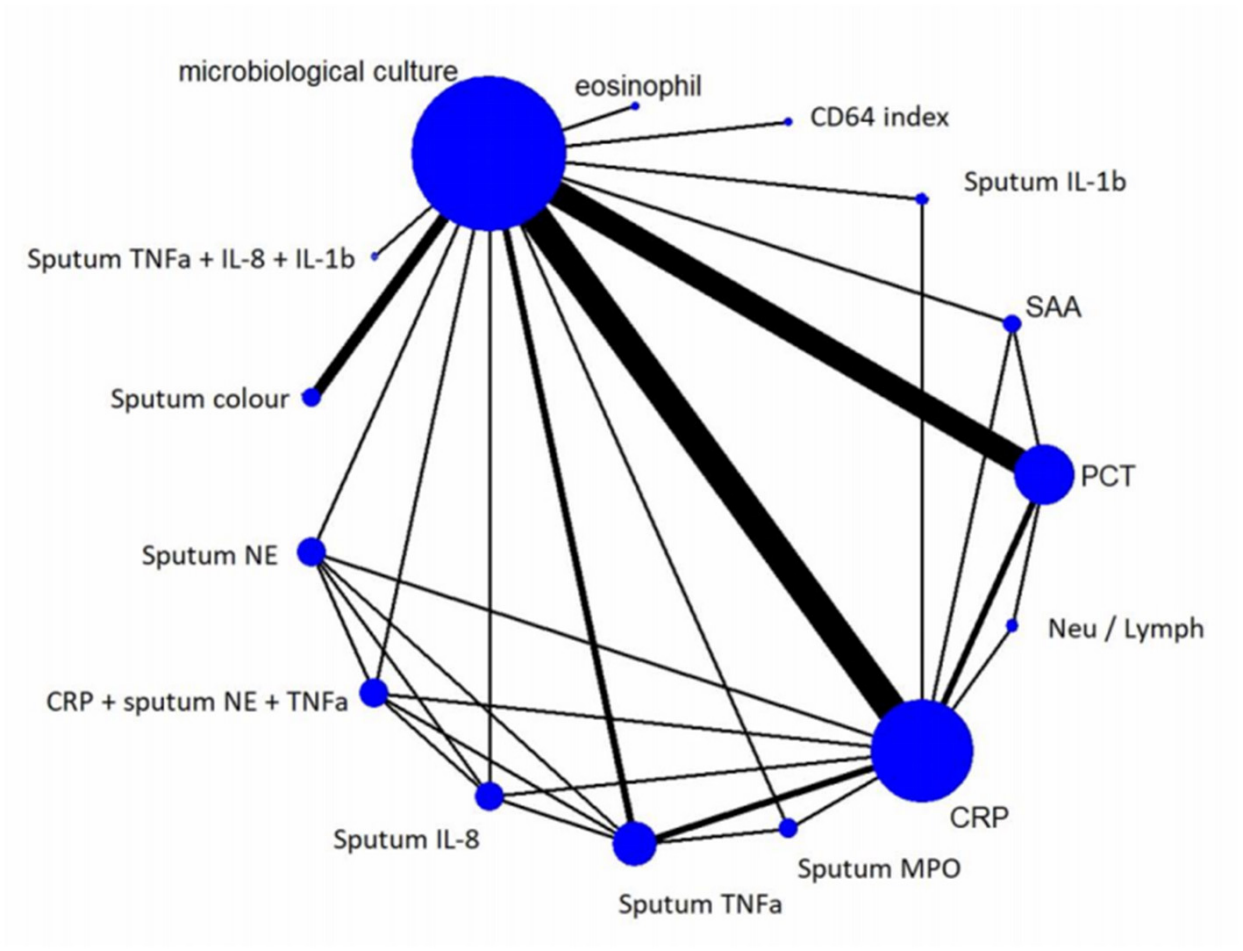
Network Graph. The network graph enables visualization of the relations between examined index tests and the reference standard. The nodes represent the tests and the edges show the direct head-to head trials. The thickness of the edges is proportional to the number of direct head-to-head trials, and the size of the nodes is proportional to the sample sizes. CRP and PCT were the most commonly investigated biomarkers for predicting bacterial infection compared to microbiological culture in AECOPD. CRP, C-reactive protein; IL-1b, interleukin one beta; IL-8, interleukin eight; MPO, myeloperoxidase; NE, neutrophil elastase; N/L, neutrophil-lymphocyte ratio; PCT, procalcitonin; SAA, serum amyloid A; TNFa, tumor necrosis factor alfa.

The network graph, shown on Figure 4 enables visualization of the relations between the 14 examined index tests and the reference standard (microbiological culture).

### Diagnostic Performance of the Index Tests

Index tests ranked by their superiority indices, and also the pooled sensitivities and specificities are depicted in Table 2. The greater the SI, the more accurate the index test is to identify the target condition of bacterial AECOPD compared to other index tests.

**Table 2:** Index tests ranked by their superiority indices. Pooled sensitivity and specificity of index tests are also shown.

**Ranking of graph**	**Index test**	**Superiority index mean (CI)**	**Pooled sensitivity mean (CI)**	**Pooled specificity mean (CI)**
#1	Sputum IL-1b	8.75 (0.05–25.00)	74% (26–97%)	65% (19–93%)
#2	Sputum TNFa	7.23 (0.07–25.00)	70% (35–96%)	70% (33–93%)
#3	Sputum TNFa+IL-8+IL-1b	7.05 (0.05–25.00)	67% (19–96%)	69% (26–98%)
#4	Sputum MPO	6.52 (0.04–25.00)	78% (32–100%)	50% (13–89%)
#5	CD64 index	5.05 (0.04–21.00)	71% (20–97%)	55% (15–88%)
#6	Sputum NE+TNFa+ serum CRP	4.23 (0.05–23.00)	68% (23–96%)	58% (17–91%)
#7	SAA	3.20 (0.04–21.05)	65% (22–96%)	55% (14–89%)
#8	Sputum IL-8	2.75 (0.04–19.00)	59% (17–92%)	58% (16–91%)
#9	Sputum NE	2.65 (0.04–19.00)	64% (20–92%)	55% (18–91%)
#10	Sputum Color	1.80 (0.05–13.00)	71% (42–90%)	51% (30–73%)
#11	Eosinophil %	1.74 (0.04–17.00)	66% (22–96%)	43% (8–87%)
#12	PCT	1.71 (0.09–9.00)	54% (39–69%)	71% (59–79%)
#13	CRP	1.70 (0.11–9.0)	67% (54–77%)	62% (52–71%)
#14	N/L	1.43 (0.04–15.00)	59% (17–89%)	51% (15–87%)

*CRP, C-reactive protein; IL-1b, interleukin one beta; IL-8, interleukin eight; MPO, myeloperoxidase; NE, neutrophil elastase; N/L, neutrophil-lymphocyte ratio; PCT, procalcitonin; SAA, serum amyloid A; TNFa, tumor necrosis factor alfa; CI, confidence interval*.

Inflammatory biomarkers isolated from sputum yielded higher rankings compared to serum biomarkers. Sputum IL-1b showed the highest SI with a pooled sensitivity of 74% (CI: 26–97) and pooled specificity of 65% (CI: 19–93). IL-1b in the lung is produced by epithelial cells and promotes the secretion of pro-inflammatory cytokines.

### Risk of Bias and Applicability Assessment

A summary of risk of bias and applicability assessment, according to QUADAS-2 is presented in the Supplementary Figures 2, 3. Patient selection domains in the majority of articles carried low or unclear risk of bias, the latter being attributed to limited reporting on inclusion and exclusion criteria. The majority of index tests were categorized as carried unclear risk of bias due to the lack of predefined thresholds for the index tests. Reference standard was found to be of low risk of bias in all but two studies where it carried high risk (9, 20) as patients with reportedly unreliable sputum microbiology were included in the analysis. Four studies (9, 17, 20, 34), were evaluated as high risk in flow and timing because a non-uniform reference standard was used, and not all the included patients were included in the analysis.

## Discussion

The aim of our study was to investigate whether on-admission inflammatory biomarkers could identify bacterial infection in AECOPD. Therefore, we evaluated serum and sputum inflammatory biomarkers and ranked them by their diagnostic performances as compared to the reference standard of microbiological culture. Our results show that although there is a statistically significant difference between PCT and CRP levels on admission between bacterial and non-bacterial AECOPD, it is important to note that these differences are most likely negligible in clinical practice, as supported by the results of DTA-NMA. Neither serum nor sputum biomarkers obtained on admission are sensitive or specific enough to accurately differentiate the bacterial etiology of AECOPD. Though our network meta-analysis suggests that sputum biomarkers are superior to serum biomarkers, they too only confirm moderate sensitivity and specificity. According to our results, the routinely used serum biomarkers; CRP and PCT were also unreliable in this regard.

Even though an increasing number of studies show that AECOPD occurs due to a variety of infectious and non-infectious etiologies, in clinical practice antibiotics are widely prescribed for AECOPD patients. Although untreated bacterial infections may cause serious complications, treating viral or non-infective causes of inflammation with antibiotics is not only ineffective, but also contributes to the development of antibiotic resistance, increases healthcare costs and adds to the risk of antibiotic associated adverse events (37). Regarding the search for a single marker for the early recognition of bacterial infection—based on previous inconsistent reports and our current analysis, one should be very cautious to utilize any on-admission inflammatory biomarker to initiate or to drive the decision to administer antibiotics.

Numerous previous studies have suggested that PCT is a specific biomarker for bacterial infections in various diseases (37–40). However, a recent meta-analysis (2), which investigated the diagnostic ability of PCT for predicting bacterial infection in AECOPD, as well as the utility of PCT guided antibiotic treatment, found that PCT measured on admission affords moderate value in diagnosing bacterial infections in AECOPD. Their findings are aligned with our results, which show that the routinely used serum biomarkers CRP and PCT confer only moderate sensitivity (67 and 54%, respectively) and moderate specificity (62 and 71%, respectively) to predict bacterial infection on admission, when compared to microbiological culture. The 2020 Report of the Global Initiative for Chronic Obstructive Lung Disease (GOLD) also states that there is only low to moderate evidence supporting PCT-guided antibiotic management in AECOPD (3). Numerous previous studies have investigated the diagnostic value of inflammatory biomarkers for differentiating bacterial infection in several conditions. With various results on reliability (37–43), PCT seems to be a good diagnostic aid to confirm the suspicion of bacterial infection in neonatal fever (40, 44), pancreatitis (41, 42) and pneumonia (45, 46), whereas CRP has only shown moderate diagnostic performance compared to PCT. However, bedside application of these findings is difficult. A closer look at any scatter plot of biomarkers comparing bacterial to viral, or infectious to non-infectious origins, shows the huge overlap between the boxplots, which is due to the heterogeneity of the host's immune response to the acute insult. It has also been emphasized in some articles that defining cut-off values for certain biomarkers for differential diagnostic purposes is a disappointing approach (46, 47). Our findings add to the evidence that this concept of using a given cut-off value for decision making at admission can be misleading.

### Implications for Clinical Practice

Current meta-analysis provides further support to the frequent observation at the bedside that, upon admission, based on a single measurement of a biomarker, clinicians cannot judge the presence of a bacterial infection in AECOPD patients with acceptable accuracy. Furthermore, measuring inflammatory biomarkers from sputum seems to be an increasingly accepted method of studying airway inflammation, and the development of a rapid, bedside-patient test would be of great use in clinical practice.

### Implications for Research

Recently published results of prospective observational studies indicate that the evaluation of the dynamic changes in biomarker levels over time may give more reliable help to the clinicians' decision making than absolute values (43, 47). Both studies found that an almost two-fold PCT increase within 24 hours was significantly associated with proven infection. Unfortunately, this approach may not be feasible in the Emergency Department and managing patients according to biomarker dynamics has not been tested in randomized controlled trials.

The differential diagnosis of the presence of bacterial infection in a chronic inflammatory condition such as COPD is no doubt a challenge, and should be determined by taking multiple factors into account. The current meta-analysis only evaluated the most commonly investigated serum and sputum biomarkers. Investigations regarding lung function in bacterial and non-bacterial AECOPD might also help in the decision of administering antibiotics. It is also important to note that, at the time of conduction of the current meta-analysis, data were scarce on emerging new biomarkers, which should be considered for future research.

### Limitations

We are aware, that the current meta-analysis has several limitations. The clinical heterogeneity of the overall population is considerable, which is due to several factors. To increase generalizability of the AECOPD population we included studies that discussed outpatient populations—both general practitioner and Emergency Department visits, and studies that discussed hospitalized patients on respiratory wards and also in intensive care units. Furthermore, heterogeneity of the patient population also limits the interpretation of the results, as inflammatory response differs with severity of disease. Therefore, investigating better specified subpopulations such as only outpatients, or hospitalized or those receiving mechanical ventilation separately could produce different results, hence further studies in these fields are warranted.

In the absence of a reference gold standard, we used the currently considered most accepted, easily obtainable test to determine the presence of bacterial infection: the microbiology results. The bacterial growth, from which a culture was considered positive, varied across the included studies. Also, the location of the microbiological samples differed between studies, which also add to the uncertainty of precise interpretation of our results; while most studies used sputum samples, studies conducted in intensive care units preferred endotracheal aspirates. In one study (17) not all patients received the same reference standard, samples were obtained for culture from sputum and endotracheal aspirates.

We are also aware that chronic bacterial colonization confounds microbiological samples taken at exacerbations. The studies included in our meta-analysis did not differentiate the potentially new strains of pathogens from the colonizing bacteria, which adds to the limitations of our analysis. Thus, the search for a reliable, rapidly available test to differentiate new bacterial infection is still to be continued.

Timing is also a matter of concern. Obtaining a sample on admission to hospital indicates a completely different trajectory to when the sampling was performed in an outpatient clinic or on admission to the intensive care unit (ICU).

It is also important to note that the majority of included studies did not pre-define cut-off values, or reported on more than one cut-off for the examined index tests. Also, unfortunately, the literature was scarce on any new, rare biomarkers currently being investigated in AECOPD. Though on hospital visit clinicians almost always evaluate full blood count, our literature search only found one study investigating the diagnostic performance of neutrophil/lymphocyte ratio and eosinophil percentage to identify exacerbations due to bacterial infections. As some serum biomarkers (SAA, CD64 index) are uncommon and the analysis of sputum biomarkers is not routinely performed at hospital admissions it is no surprise that literature was insufficient on these biomarkers.

Referring to previously mentioned studies and our meta-analysis the potential of a single biomarker determined on admission only holds moderate diagnostic value. However, investigating combinations of biomarkers might increase the diagnostic ability and accuracy to identify new bacterial infections. Literature was also scarce on volatile biomarkers that might also hold some potential in the question.

## Methods

### Protocol and Registration

This DTA-NMA is reported according to the Preferred Reporting Items for Systematic Review and Meta-Analysis of Diagnostic Test Accuracy Studies (PRISMA-DTA) Statement (48). The protocol of the meta-analysis was registered on PROSPERO before data collection under registration number CRD42020161301.

### Data Source and Search Strategy

A systematic literature search was performed in five databases: MEDLINE via PubMed, Embase, CENTRAL, Scopus and Web of Science in October 2019. Detailed search strategy is shown in the Supplementary Table 1.

### Eligibility, Selection, and Data Collection

Studies that reported: a) adult patients with bacterial and non-bacterial AECOPD; b) results of microbiology tests (as the reference standard) with samples taken from sputum, tracheal aspirates or blood; and c) at least one other on-admission diagnostic test performed from serum or sputum (index tests), were considered eligible. The index tests included C-reactive protein (CRP), procalcitonin (PCT), white blood cell count (WBC), neutrophil/lymphocyte ratio (N/L), eosinophil percentage (eo %), or serum amyloid A (SAA). As sputum biomarkers, neutrophil elastase (NE), tumor necrosis factor alfa (TNFa), myeloperoxidase (MPO), and interleukins were included. Studies assessing the diagnostic ability of sputum color were also included.

We excluded studies that did not discriminate AECOPD from pneumonia, other acute or chronic lung diseases, or other extrapulmonary sources of infection, or underlying conditions that could influence respiratory function or inflammatory biomarker levels (e.g., cardiac related conditions, chronic immunosuppression). We also excluded studies that only assessed sputum quality or culture contamination rates. Detailed description of eligibility criteria is reported in the Supplementary Material. Details of included studies can be found in Table 1 and Supplementary Tables 2, 3.

Prospective or retrospective cross-sectional studies were selected for inclusion. Both conference abstracts and full-text articles were included. Conference abstracts were only included if it provided information on patient inclusion and exclusion criteria, if stated the use of on-admission microbiological culture as reference standard, and if provided information on the diagnostic accuracy of index tests. In cases of suspected overlapping study populations, we preferred to include full-text articles over abstracts and greater sample size over smaller.

Two independent review authors completed all steps of selection and data collection (PP, DD) into a pre-defined data collection sheet in duplicate. Any disagreements were resolved by involving an independent third party (ZS). Further information on selection and data extraction are detailed in the Supplementary Material.

### Risk of Bias and Applicability

Risk of bias and applicability within the included studies were assessed by two review authors (PP, DD) according to the Quality Assessment of Diagnostic Accuracy Studies-2 (QUADAS-2) tool. Any disagreement was resolved by an independent third party (ZS).

### Statistical Analysis

#### Statistical Analysis of Weighted Mean Difference

We calculated the differences in the levels of CRP, PCT and WBC between bacterial and non-bacterial AECOPD groups. For these parameters we investigated the WMD with a 95% confidence interval (CI). Pooled estimates were calculated with a random effects model by using the DerSimonian–Laird method (49). Results of the meta-analysis were displayed graphically using Forest plots. Heterogeneity was tested by using the Cochrane's Q and the *I*^2^ statistics, where *I*^2^ = 100% × (Q–df)/Q, and represents the magnitude of the heterogeneity (moderate: 30–60%, substantial: 50–90%, considerable: 75–100%) (50). These meta-analytical calculations were performed by Stata 11 data analysis and statistical software (Stata Corp LLC, College Station, TX, USA).

#### Statistical Analysis of Diagnostic Test Accuracy Network Meta-Analysis

We created 2 × 2 contingency tables according to the diagnostic accuracy tests, which describe the relationship between the results of the index test and the reference standard at a given diagnostic threshold. Target condition was bacterial exacerbation. A positive microbiological culture was used to confirm a bacterial exacerbation. We used the number of documented exacerbations with positive and negative culture results. The tables include the number of true positives (TP), false positives (FP), false negatives (FN), and true negatives (TN). If more than one cut-off value was reported for the same index test, we chose the best cut-off. We also collected the data on numbers of patients with bacterial and non-bacterial etiology of AECOPD. We performed a DTA-NMA to investigate the diagnostic abilities of serum and sputum inflammatory biomarkers on differentiating bacterial infection in AECOPD. To assess the relative performance of a diagnostic test, we calculated pooled sensitivity and pooled specificity for the index tests compared to microbiological culture and ranked them based on the superiority index (SI). The greater the SI, the greater the accuracy of the screening test to identify the target condition compared to other screening tests. The network was depicted in a graph, with the nodes representing the different screening methods and the edges representing head-to-head comparisons. The size of the node correlates with the number of studies. The thickness of the edges correlates with the number of trials with a head-to-head comparison. All statistical calculations were performed using R programming language (v3.6.1) with rstan, loo and plyr packages. Data of contingency tables from each study is presented in the Supplementary Table 2.

## Conclusion

Clinicians cannot rely on the routinely used inflammatory biomarkers determined from serum or sputum obtained on admission to predict the presence of bacterial infection in AECOPD with high certainty.

## Data Availability Statement

The original contributions presented in the study are included in the article/Supplementary Material, further inquiries can be directed to the corresponding author/s.

## Author Contributions

PP and DD performed the database search, read the articles for eligibility, collected the data from the articles to the study database, and performed the bias analysis and quality assessment, when a conflict arose, a third participant, ZS made the decision. Statistical analysis was conducted by AS. ZS helped interpreting the analysis. PP and ZM drafted the manuscript. PP, AP, ZS, and ZM edited the manuscript. PP edited the tables and figures and completed the PRISMA checklist. ZM made the critical revision on the finalized manuscript. All authors reviewed and approved the final manuscript.

## Funding

The article was funded by the project titled GINOP-2.3.2-15-2016-00048 - STAY ALIVE is co-financed by the European Union (European Regional Development Fund) within the framework of Programme Széchenyi 2020 and Human Resources Development Operational Programme Grant, Grant Number: EFOP-3.6.2-16-2017-00006 – LIVE LONGER is co-financed by the European Union (European Regional Development Fund) within the framework of Programme Széchenyi 2020.

## Conflict of Interest

The authors declare that the research was conducted in the absence of any commercial or financial relationships that could be construed as a potential conflict of interest.

## Publisher's Note

All claims expressed in this article are solely those of the authors and do not necessarily represent those of their affiliated organizations, or those of the publisher, the editors and the reviewers. Any product that may be evaluated in this article, or claim that may be made by its manufacturer, is not guaranteed or endorsed by the publisher.

## Supplementary Material

Supplementary Figure 1 Forest plot of white blood cell count. WBC was measured in AECOPD cases with bacteria positive and bacteria negative microbiological cultures. WBC was reported in (1000/ml) in each study. WMD = 1.07, 95% CI: 0.40961.74, (p = 0.002). AECOPD, acute exacerbation of chronic obstructive pulmonary disease; WBC, white blood cell count; SD, standard deviation; WMD, weighted mean difference; CI, confidence interval.

Supplementary Figure 2 Methodological Quality Assessment of DTA-NMA. The graph shows the summary of methodological quality assessment of studies according to Quality Assessment of Diagnostic Accuracy Studies-2 (QUADAS-2) tool concerning risk of bias and applicability in review authors' judgments about each domain (patient selection, index tests, reference standard and flow and timing) for each included study. The second column shows review authors' judgments about each domain, presented as percentages across included studies. Green color represents low risk, yellow represents unclear risk, and red represents high risk of bias. CRP, C-reactive protein; IL-1b, interleukin one beta; IL-8, interleukin eight; MPO, myeloperoxidase; NE, neutrophil elastase; N/L, neutrophil-lymphocyte ratio; PCT, procalcitonin; SAA, serum amyloid A; TNFa, tumor necrosis factor alfa.

Supplementary Figure 3 Summary of Methodological Quality Assessment. The graph shows the summary of methodological quality assessment of each study according to each domain of QUADAS-2 tool. The domains of quality assessment include patient selection, index tests, reference standard, flow and timing. Green color with “+” sign represent low risk, yellow color with “?” represent unclear risk and red color with “-” represent high risk of bias. CRP, C-reactive protein; IL-1b, interleukin one beta; IL-8, interleukin eight; MPO, myeloperoxidase; NE, neutrophil elastase; N/L, neutrophil-lymphocyte ratio; PCT, procalcitonin; SAA, serum amyloid A; TNFa, tumor necrosis factor alfa.

## Abbreviations

AECOPD: Acute Exacerbation of Chronic Obstructive Pulmonary Disease
COPD: Chronic Obstructive Pulmonary Disease
GOLD: Global Initiative for Chronic Obstructive Lung Disease
CRP: C-reactive Protein
PCT: Procalcitonin
TNFa: Tumor necrosis factor alfa
SAA: Serum amyloid A
MPO: Myelo-peroxidase
IL-8: Interleukin-8
IL-1b: Interleukin-1-beta
NE: Neutrophil Elastase
ED: Emergency Department
ICU: Intensive Care Unit
QUADAS-2: Quality Assessment of Diagnostic Accuracy Studies-2
TP: True positive
FP: False positive
TN: True negative
FN: False negative
DTA: Diagnostic Accuracy
NMA: Network Meta-analysis
WMD: Weighted Mean Difference
CI: Confidence Interval
SD: Standard Deviation
IQR: Interquartile Range.
