# Supplementary figures and images for: Inflammatory Biomarkers Are Inaccurate Indicators of Bacterial Infection on Admission in Patients With Acute Exacerbation of Chronic Obstructive Pulmonary Disease—A Systematic Review and Diagnostic Accuracy Network Meta-Analysis
Source: Front Med (Lausanne). 2021 Nov 18;8:639794. doi: 10.3389/fmed.2021.639794 (PMC8636902; doi:10.3389/fmed.2021.639794)

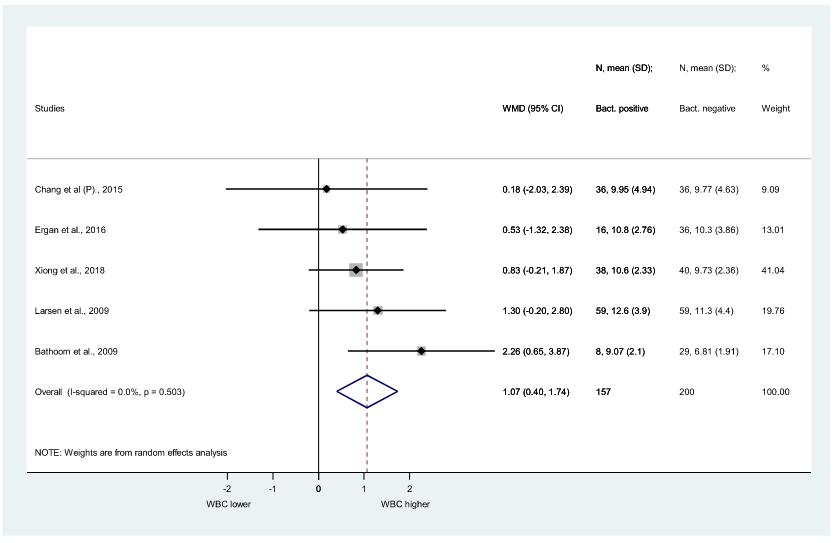

Supplement: Supplementary Figure 1 — Forest plot of white blood cell count. WBC was measured in AECOPD cases with bacteria positive and bacteria negative microbiological cultures. WBC was reported in (1000/ml) in each study. WMD = 1.07, 95% CI: 0.40961.74, (p = 0.002). AECOPD, acute exacerbation of chronic obstructive pulmonary disease; WBC, white blood cell count; SD, standard deviation; WMD, weighted mean difference; CI, confidence interval. [file Image_1.TIFF]

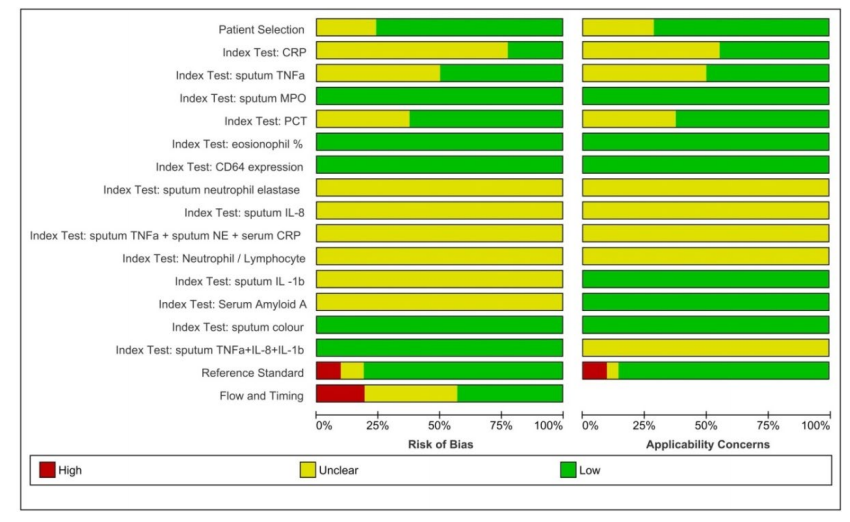

Supplement: Supplementary Figure 2 — Methodological Quality Assessment of DTA-NMA. The graph shows the summary of methodological quality assessment of studies according to Quality Assessment of Diagnostic Accuracy Studies-2 (QUADAS-2) tool concerning risk of bias and applicability in review authors' judgments about each domain (patient selection, index tests, reference standard and flow and timing) for each included study. The second column shows review authors' judgments about each domain, presented as percentages across included studies. Green color represents low risk, yellow represents unclear risk, and red represents high risk of bias. CRP, C-reactive protein; IL-1b, interleukin one beta; IL-8, interleukin eight; MPO, myeloperoxidase; NE, neutrophil elastase; N/L, neutrophil-lymphocyte ratio; PCT, procalcitonin; SAA, serum amyloid A; TNFa, tumor necrosis factor alfa. [file Image_2.TIFF]

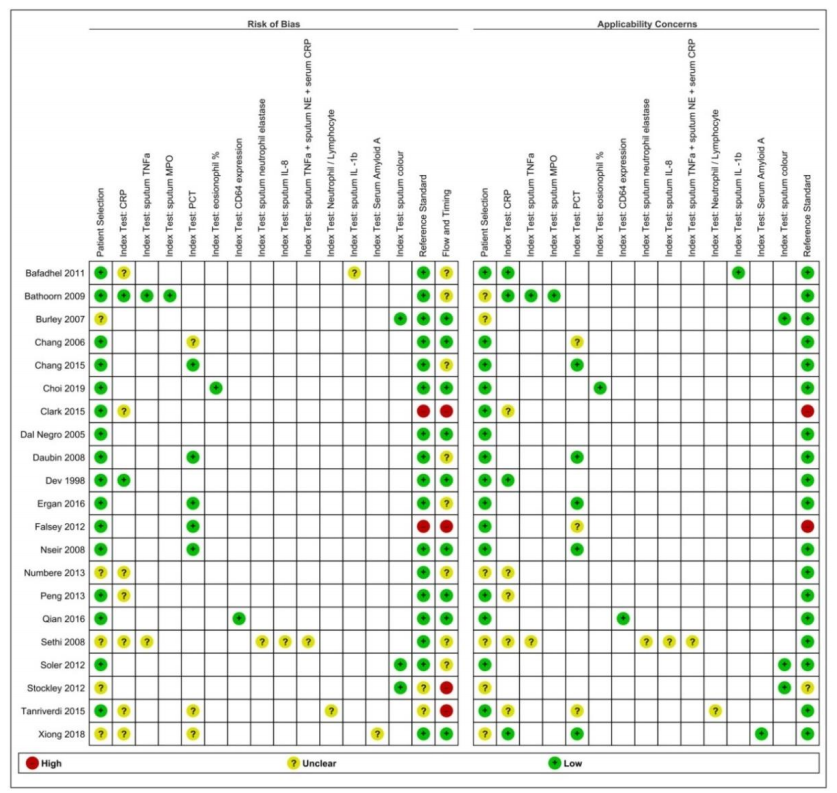

Supplement: Supplementary Figure 3 — Summary of Methodological Quality Assessment. The graph shows the summary of methodological quality assessment of each study according to each domain of QUADAS-2 tool. The domains of quality assessment include patient selection, index tests, reference standard, flow and timing. Green color with “+” sign represent low risk, yellow color with “?” represent unclear risk and red color with “-” represent high risk of bias. CRP, C-reactive protein; IL-1b, interleukin one beta; IL-8, interleukin eight; MPO, myeloperoxidase; NE, neutrophil elastase; N/L, neutrophil-lymphocyte ratio; PCT, procalcitonin; SAA, serum amyloid A; TNFa, tumor necrosis factor alfa. [file Image_3.TIFF]
