# Supplementary material for: Inflammatory Biomarkers Are Inaccurate Indicators of Bacterial Infection on Admission in Patients With Acute Exacerbation of Chronic Obstructive Pulmonary Disease—A Systematic Review and Diagnostic Accuracy Network Meta-Analysis
Source: Front Med (Lausanne). 2021 Nov 18;8:639794. doi: 10.3389/fmed.2021.639794 (PMC8636902; doi:10.3389/fmed.2021.639794)
Supplement: Supplementary file 4 [file Data_Sheet_1.docx]

**SUPPLEMENTARY MATERIAL**

**Detailed Search Strategy**
A systematic literature search was conducted in five databases with the following search-key:
(((COPD OR 'chronic obstructive lung disease' OR 'chronic obstructive pulmonary disease')) AND (exacerbation OR AECOPD)) AND (PCT OR procalcitonin OR CRP OR c reactive protein OR lymphocyte OR neutrophil OR interleukin-6 OR IL-6 OR TNF alfa OR tumor necrosis factor alfa)

No filters were used. The date of search was: 15. 10. 2019.
*Supp. Tabl. 1.* presents results of systematic search in each database

| **Database** | **Date of Search** | **Number of records** |
| --- | --- | --- |
| MEDLINE (Pubmed) | 2019.10.15. | 900 |
| Embase | 2019.10.15. | 2489 |
| Scopus | 2019.10.15. | 2286 |
| Cochrane Library | 2019.10.15. | 459  (174 reviews, 285 trials) |
| Web of Science | 2019.10.15. | 1448 |
| **Total** | 2019.10.15. | **7582** |

1. Supp. Table Search Strategy

**Protocol Deviation**

Our protocol was registered as a study assessing the weighted mean difference (WMD) of inflammatory biomarkers in infectious and non-infectious etiologies of AECOPD. Instead of WMD analysis, investigating the diagnostic accuracy of inflammatory biomarkers and comparing their diagnostic performances in a DTA-NMA seemed a better approach to evaluate the clinical role of biomarkers in differentiating bacterial and non-bacterial AECOPD. To comply with the previously registered protocol, we also completed WMD analysis of serum biomarkers. Results are shown in the Additional Analysis in the text and in the *Supplementary Material.*

**Detailed Study Selection**After the conduction of literature search we imported all 7582 items found in previously mentioned databases into EndNote X7.8 (Clarivate Analytics, Philadelphia, USA) for screening and selection. After duplicate removal, 3869 items remained for further selection. Screening by titles was done by two independent researchers; PP and DD. Items were included if title mentioned COPD or AECOPD and/or mention of any inflammatory biomarkers or if title referred to target condition.
Abstracts were excluded if the article compared AECOPD to stable state of COPD, if there was no mention of infective / non-infective, or bacterial / non-bacterial etiology or microbiological culture or no mention of measured inflammatory biomarkers. After abstract selection, 70 full texts were eligible for assessment.
Full texts were excluded if the study compared pneumonia with AECOPD (6 articles), if study compared AECOPD with stable state of COPD (8 articles) if the article did not report microbiological culture for identifying bacterial etiology of exacerbation (4 articles), if there was only qualitative report on microbiology without measure of inflammatory biomarkers (3 articles) or if study did not publish relevant data either according to diagnostic accuracy testing or according to weighted mean difference measurements of biomarker levels. Finally 21 items were eligible for inclusion in the diagnostic network meta-analysis, and also 22 items were eligible for analysis according to weighted mean differences of biomarkers. *Fig. 1.* shows the PRISMA flowchart of selection process.

**Detailed Data Extraction**Data collection was done by two independent researchers (PP and DD) on a previously defined data sheet. Data was collected as to create 2x2 contingency tables for diagnostic accuracy testing. We collected data based on diagnostic metrics, such as sensitivity, specificity, number of exacerbations with positive microbiological culture, and biomarker level above cut-off value defined by each study (true positives: TP), exacerbations with negative microbiology, but biomarker level above cut-off value (false positives: FP), exacerbations with positive microbiology, but biomarker level below cut-off value (false negatives: FN) and exacerbations with negative microbiology and biomarker level below cut-off value (true negatives: TN), positive predictive value (PPV) and negative predictive value (NPV). Where the study did not report data on 2x2 tables, they were calculated, if possible from the reported data on the binary classification tests. Data of the 2x2 contingency tables is presented on *Supp. Tabl. 2.*

On each study; author, year, country, number of study centers, study design, population (inclusion and exclusion criteria), reference standard, index tests and their cut-off values, sensitivity and specificity were extracted into the data sheet.
Also data reported on the biomarkers’ measured mean, standard deviation (SD), median, interquartile range (IQR), range were extracted into the data table for weighted mean difference analysis. Any disagreements on the data extraction were solved by discussion or by an independent third party (ZS).

| **Author, Year of Publication** | **Reference standard (microbiological culture obtained from)** | **Index tests (cut-off value)** | **Sample size (n)** | **Bacterial (n)** | **TP** | **FP** | **FN** | **TN** |
| --- | --- | --- | --- | --- | --- | --- | --- | --- |
| **Tanriverdi et al. 2015 [19]** | tracheal aspirate or sputum | CRP (91.5 mg/l) | 77 | 28 | 15 | 24 | 13 | 25 |
|  |  | PCT (0.4 ng/ml) |  |  | 17 | 16 | 11 | 33 |
|  |  | N/L (11.5) |  |  | 17 | 24 | 11 | 25 |
| **Dev et al. 1998 [32]** | sputum | CRP (10 mg/l) | 50 | 29 | 29 | 13 | 0 | 8 |
| **Peng et al. 2013 [33]** | sputum | CRP (19.65 mg/l) | 81 | 55 | 43 | 4 | 12 | 22 |
| **Clark et al. 2015 [18]** | blood, sputum, urine and nasopharyngeal swab | CRP (10 mg/l) | 264 | 66 | 45 | 70 | 21 | 128 |
| **Xiong et al. 2018 [34]** | sputum | CRP (31.68 mg/l) | 78 | 28 | 26 | 6 | 12 | 34 |
|  |  | PCT (0.76 ng/ml) |  |  | 30 | 3 | 8 | 37 |
|  |  | SAA (31.28 mg/l) |  |  | 32 | 10 | 6 | 30 |
| **Sethi et al. 2008 [35]** | sputum | CRP (2.37 mg/l) | 150 exacerbations from 46 patients | 39 | 24 | 35 | 15 | 76 |
|  |  | sputum NE (0.76 nM) |  |  | 28 | 36 | 11 | 75 |
|  |  | sputum IL-8 (1.39 ng/ml) |  |  | 26 | 38 | 13 | 73 |
|  |  | sputum TNFa (320 pg/ml) |  |  | 28 | 33 | 11 | 78 |
| **Bathoorn et al. 2009 [36]** | sputum | CRP (2 mg/l) | 37 | 8 | 7 | 17 | 1 | 12 |
|  |  | sputum TNFa (30pg/ml) |  |  | 8 | 3 | 0 | 26 |
|  |  | sputum MPO (12 ug/ml) |  |  | 8 | 15 | 0 | 14 |
| **Numbere et al. 2013 [37]** | sputum | CRP (50 mg/l) | 122 | 67 | 33 | 23 | 34 | 32 |
| **Bafadhel et al. 2011 [38]** | sputum | CRP (10 mg/l) | 148 exacerbations (75 patients) | 79 | 47 | 21 | 32 | 48 |
|  |  | sputum IL-1b (125 pg/ml) |  |  | 71 | 14 | 8 | 55 |
| **Nseir et al. 2008 [39]** | endotracheal aspirate | PCT (0.5 ng/ml) | 98 | 40 | 18 | 17 | 22 | 41 |
| **Ergan et al. 2016 [40]** | sputum or endotracheal aspirate or bronchoalveolar | PCT (0.25 ng/ml) | 52 | 16 | 10 | 12 | 6 | 24 |
| **Falsey et al. 2012 [9]** | blood culture, sputum Gram stain and culture, nose and throat swabs | PCT (0.25 ng/ml) | 184 | 32 | 8 | 23 | 24 | 129 |
| **Chang et al. 2015 [41]** | sputum | PCT (0.5 ng/ml) | 72 | 30 | 3 | 4 | 27 | 38 |
| **Chang et al. 2006 [42]** | sputum | PCT (0,155 ng/ml) | 45 | 15 | 14 | 12 | 1 | 18 |
| **Daubin et al. 2008 [43]** | sputum or tracheal aspirate | PCT (0,1 ng/ml) | 35 | 5 | 2 | 19 | 3 | 11 |
| **Choi et al. 2019 [44]** | blood, sputum and urine | eosinophil (2%) | 736 | 307 | 255 | 291 | 52 | 138 |
| **Qian et al.**  **2016 [45]** | sputum | CD64 index (2,5) | 150 | 82 | 69 | 28 | 13 | 40 |
| **Soler et al. 2012 [46]** | sputum | sputum colour (purulent) | 41 | 14 | 12 | 18 | 2 | 9 |
| **Burley et al. 2007 [47]** | sputum | sputum colour (purulent) | 97 | 58 | 35 | 13 | 23 | 26 |
| **Stockley et al. 2000 [20]** | sputum | sputum colour (purulent) | 121 | 86 | 73 | 14 | 13 | 21 |
| **Dal Negro et al. 2005 [48]** | sputum | sputum TNFa+IL-8+IL-1b (492 pg/ml, 4,81 ng/ml, 2818 pg/ml) | 124 | 48 | 37 | 5 | 11 | 71 |

**Supp. Table 2**. This table presents the results of diagnostic accuracy testing as reported in each included study.

**Characteristics of studies included into Additional Analysis**Altogether 22 studies were included in the analysis of weighted mean differences. Among studies examining the weighted mean differences of inflammatory biomarkers, the most commonly investigated biomarkers were CRP; included in 16 studies:[1, 18, 19, 32-34, 36, 39-41, 50-55] and PCT; included in 15 studies: [1, 9, 19, 34, 39-43, 45, 50, 52, 55-57] There were 5 studies reporting data on measured WBC counts: [34, 36, 40, 41, 51] *Supp. Tabl. 3*. presents characteristics of studies included in WMD analysis.

**Results of Additional Analysis***Supp. Fig. 1.* shows the Forest plot of WBC (measured in 1000/ml in each study) of bacterial and non-bacterial AECOPD. WBC was significantly higher in bacterial than in non-bacterial AECOPD. WMD=1.07, 95% CI: 0.40–1.74 (p=0.002).

**Risk of bias and applicability assessment**Risk of bias analysis was done according to the QUADAS-2 tool. The QUADAS-2 tool included the following 4 domains: patient selection, index test, reference standard and flow of patients through the study and the timing of index tests and reference standard (flow and timing). The Review Manager 5 (RevMan 5.2.3, Cochrane Collaboration, Oxford, UK) statistical computing software was used to carry out the quality assessment and investigation of publication bias. *Supp. Fig. 2. and 3.* show the Methodological Quality Assessment graph and summary.

Reviewer judgment of methodological quality of each individual study included in the analysis was performed using the Quality Assessment of Diagnostic Accuracy Studies (QUADAS-2) tool. " - " in red and " + " in green mean high risk and low risk respectively, and " ? " in yellow means unclear risk. In the index test part, most studies were valued unclear risk, as these studies did not predefine threshold. Two studies were valued high risk in the reference standard part, due to the inclusion of unreliable sputum samples into the analysis [9, 18]. Four studies [9, 18-20] were evaluated high risk in flow and timing part because of non-uniform reference standard was used and not all included patients were included in the analysis.

| **Author, Year of Publication** | **Country** | **Study centers and design*** | **Population  (inclusion, recruitment period  and exclusion)** | | **Timing of sample collection** | **Reference standard (microbiological culture obtained from)** | **Measured inflammatory biomarker** | **Sample size (bacterial %)** |
| --- | --- | --- | --- | --- | --- | --- | --- | --- |
| **Tanriverdi et al. 2015 [19]** | Turkey | single center prospective cohort | hospitalized AECOPD patients Jan 01 – March 31 2014 | pneumonia | admission | tracheal aspirate or sputum | CRP PCT | 77 (36) |
| **Dev et al. 1998 [32]** | United Kingdom | prospective cohort | hospitalized AECOPD patients  (recruitment period not reported) | pneumonia | admission | sputum | CRP | 50 (58) |
| **Peng et al. 2013 [33]** | China | single center prospective cohort | hospitalized AECOPD patients (recruitment period not reported) | infiltrate on x-ray, asthma, bronchiectasis, malignancy, inflammatory diseases | admission | sputum | CRP | 81 (68) |
| **Clark et al. 2015 [18]** | United Kingdom | single center retrospective analysis | hospitalized AECOPD patients 2005-2008 between Sept-May | alternate diagnosis, pneumonia, cardiovascular disease | admission | blood, sputum, urine and nasopharyngeal swab | CRP | 264 (25) |
| **Xiong et al. 2018 [34]** | China | single center prospective cohort | hospitalized AECOPD patients Jan 2014 - Jan-2016 | severe organ disfunction, systemic disease, malignancy | admission | sputum | CRP  PCT  WBC | 78 (49) |
| **Lacoma et al. 2011 [50]** | Spain | two centers prospective cohort | hospitalized AECOPD patients 2001 Sept-2005 Sept | asthma, cystic fibrosis, tuberculosis | not reported | sputum | CRP PCT | 161 (47) |
| **Bathoorn et al. 2009 [36]** | Netherlands | single center prospective cohort | COPD outpatients experiencing an exacerbation (recruitment period not mentioned) | immunosuppression, asthma, long term oxygen therapy, other interfering disease | at exacerbation visit | sputum | CRP WBC | 37(22) |
| **Larsen et al. 2009 [51]** | Denmark | single center prospective cohort | hospitalized AECOPD patients  Jan 01 – Dec 31 2004 | pneumonia | not reported | sputum | CRP WBC | 118 (50) |
| **Daniels et al. 2010 [52]** | Netherlands | two centers randomized controlled trial | hospitalized AECOPD patients  Aug 2002 – Feb 2008 | fever, prior antibiotic treatment, extensive treatment with corticosteroids, pneumonia, history of requiring mechanical ventilation in previous AECOPD, other infectious disease | not reported | sputum | CRP PCT | 243 (58) |
| **Nseir et al. 2008 [39]** | France | single center prospective cohort | hospitalized AECOPD patients, requiring mechanical ventilation  Dec 2004 - June 2006 | malignancy, other reason for respiratory failure | ICU admission, before AB therapy | endotracheal aspirate | CRP PCT | 98 (41) |
| **Ergan et al. 2016 [40]** | Turkey | single center prospective cohort | hospitalized AECOPD patients, requiring ICU admission May 01 2007 – July 31 2009 | other reason for respiratory failure, ICU stay <24 hours | ICU admission | sputum or endotracheal aspirate or bronchoalveolar | CRP PCT WBC | 52 (31) |
| **Falsey et al. 2012 [9]** | USA | single center prospective cohort | hospitalized AECOPD patients with admitting diagnosis of acute respiratory tract infection Between Nov 01-May 30, 2008-2009 and 2009-2010 | aspiration, prior antibiotic therapy, immunosuppression, cavitating lung disease, conditions known to increase PCT | at enrollment | blood culture, sputum Gram stain and culture, nose and throat swabs | PCT | 184 (17) |
| **Chang et al. 2015 [41]** | Taiwan | single center prospective cohort | COPD patients who visited ER with acute exacerbation Apr 2009 – Aug 2010 | asthma, other respiratory disease | at enrollment | sputum | CRP PCT WBC | 72 (42) |
| **Chang et al. 2015 [53]** | China | single center prospective cohort | hospitalized AECOPD patients  Apr 01 2009 – Sept 30 2011 | asthma, bronchiectasis, pneumonia, cancer, sleep apnoea, other active lung disease, alternate admission diagnosis, immunosuppression, need for ICU admission | at admission | sputum | CRP | 136 (31) |
| **Chang et al. 2006 [42]** | China | single center prospective cohort | COPD outpatients with acute exacerbation (recruitment period not reported) | other chronic respiratory disease, illness >5 days, pneumonia, bacterial infection outside respiratory system | not reported | sputum | PCT | 45 (33) |
| **Daubin et al. 2008 [43]** | France | single center prospective cohort | critically ill, hospitalized AECOPD patients Sept 2005 – Sept 2006 | other causes of respiratory failure | ICU admission | sputum or tracheal aspirate | PCT | 35 (14) |
| **Gallego et al. 2016 [54]** | Spain | single center prospective cohort | COPD outpatients (recruitment period not reported) | <40 years old, asthma, cystic fibrosis, bronchiectasis, immunosuppression, pneumonia | at exacerbation visit | sputum | CRP | 300 (56) |
| **Qian et al.**  **2016 [45]** | China | single center randomized controlled trial | hospitalized AECOPD patients Jan – Dec 2014 | prior antibiotic therapy, immunosuppression, pneumonia, death from other causes, bacteria infection outside respiratory system, major trauma / surgery, other chronic respiratory diseases, cardiovascular diseases, AECOPD within 2 months | admission | sputum | PCT | 150 (55) |
| **Kawamatawong et al. 2017 [1]** | Thailand | single center cross sectional study | AECOPD patients at Emergency Department visit (recruitment period not reported) | not reported | not reported | sputum | CRP PCT | 62 (47) |
| **Fu et al. 2019 [55]** | China | single center prospective cohort | hospitalized AECOPD patients  Sept 2016 – May 2018 | pneumonia, other pulmonary disease, malignancy, immunosuppression, prior antibiotic therapy, admitted 1 week before AECOPD episode, required mechanical ventilation, other systemic disease | not reported | sputum | CRP PCT | 68 (44) |
| **Rammert et al. 2009 [56]** | France | single center prospective cohort | hospitalized AECOPD patients requiring mechanical ventilation | malignancy, other cause of respiratory failure, pneumonia, bronchiectasis | before antibiotic treatment | endotracheal aspirate | PCT | 116 (36) |
| **Hassaan et al. 2012 [57]** | Saudi Arabia | single center prospective cohort congress abstract | AECOPD patients | not reported | on presentation | sputum | PCT | 50 (40) |

Supp. Table 3. Characteristics of studies included in WMD analysis. WMD: weighted mean difference
